# Costs and Benefits of Wax Production in the Larvae of the Ladybeetle *Scymnus nubilus*

**DOI:** 10.3390/insects12050458

**Published:** 2021-05-16

**Authors:** Paulo Pacheco, Isabel Borges, Beatriz Branco, Eric Lucas, António Onofre Soares

**Affiliations:** 1 Faculty of Sciences and Technology, University of the Azores, Rua da Mãe de Deus, 13-A, 9501-321 Ponta Delgada, Portugal; alexandre_biotech@hotmail.com (P.P.); bgbranco@hotmail.com (B.B.); 2 cE3c-ABG-Centre for Ecology, Evolution and Environmental Changes and Azorean Biodiversity Group, Faculty of Sciences and Technology, University of the Azores, 9501-321 Ponta Delgada, Portugal; isabel.mm.borges@uac.pt; 3 Laboratoire de Lutte Biologique, Département des Sciences Biologiques, Université du Québec à Montréal, C.P. 8888 Succursale Centre-Ville, Montreal, QC H3C 3P8, Canada; lucas.eric@uqam.ca

**Keywords:** coccinellid larvae wax cover, defensive strategy, trade-offs, intraguild predation, biological control

## Abstract

**Simple Summary:**

*Scymnus nubilus* Mulsant (Coleoptera: Coccinellidae) is a tiny ladybird reaching a maximum body weight of about 1.5 mg. Despite its small body size, the individuals of this species are able to thrive in aphidophagous guilds with other predator species with stronger competitive abilities and potential to exert intraguild predation (IGP). In this study, we explore to what extent that the wax layer of *S. nubilus* larvae produced by dorsal epidermal cells is an effective defensive mechanism. We predict that wax production by larvae is a trait selected by adaptive evolution where some benefits (eventually protection against intraguild predation) were traded with some costs. In manipulative experiments, we found that waxless *S. nubilus* larvae (waxes removed artificially) were more susceptible to IGP by lacewing larvae of *Chrysoperla agilis* (Neuroptera: Chrysopidae). We also found that adults originating from waxless larvae were lighter than the ones originating from wax larvae, demonstrating a metabolic cost resulting from a constant need of wax production. The results indicate the potential existence of a trade-off between growth and protection associated with wax production in beetles.

**Abstract:**

BACKGROUND: Larvae of the minute aphidophagous *Scymnus nubilus* Mulsant (Coleoptera: Coccinellidae) are common predators in apple orchards, covered by a wax layer that might act as a defense mechanism against natural enemies. However, the costs and benefits of protection conferred by wax remain to be assessed. We tested the following hypothesis: there is a trade-off in wax producing ladybeetles between the protection conferred by wax cover and the physiological or behavioral costs associated with its production. We predict that: (1) wax production is an efficient defensive mechanism (against intraguild predation), (2) wax production is associated with detrimental physiological (growth, reproduction) or behavioral effects (behavioral compensation: increased biomass consumption). RESULTS: Tests were carried out in the laboratory with wax and waxless larvae of *S. nubilus,* with and without lacewing larvae of *Chrysoperla agilis* (Neuroptera: Chrysopidae) being used as a potential intraguild predator of the coccinellid. Waxless individuals were more susceptible to intraguild predation by lacewing larvae. Adults originating from waxless larvae were lighter than the ones originating from wax larvae, suggesting a metabolic cost resulting from a constant need of wax production. Body-weight gain and conversion efficiency were lower in waxless larvae. Biomass consumption was similar, showing that waxless larvae did not compensate for the physiological cost by eating more aphid biomass. CONCLUSION: The results indicate the potential existence of a trade-off between growth and protection associated with wax production.

## 1. Introduction

Wax production is a common defensive strategy used by plants [1], herbivores [2,3] and predators [4,5] to reduce their susceptibility to their natural enemies. Furthermore, predators may indirectly appropriate wax from other producers. Larvae of the debris-carrying green lacewing *Ceraeochrysa lineaticornis* (Fitch) (Neuroptera: Chrysopidae) incorporate in their dorsum packets the waxy flocculence and exuviae of flatid planthopper [6].

Among coccinellids, beneficial predators in biocontrol programs [7,8,9], the different species are protected by an array of chemical, behavioural, and/or morphological defense mechanisms evolved against predation and parasitism [10,11]. Reflex bleeding is an example of a chemical defense in which coccinellid larvae and adults emit distasteful and toxic droplets of hemolymph [12]. A common behavior in predators exploiting ant-attending aphid colonies is to cover their body with dead aphid carcasses to deter ant attacks, as documented in the cecidomyid *Aphidoletes aphidimyza* Rondani (Diptera: Cecidomyiidae) [13], and the lacewing *Mallada desjardinsi* Navas (Neuroptera: Chrysopidae) [14]. Another defensive mechanism involves the production of a wax covering, such as in the larvae of *Scymnus* spp., which provides protection against predation and ant aggression [4,15]. Chemical cues are also associated with waxes. Contact chemical cues perceived by females of *Cryptolaemus montrouzieri* Mulsant (Coleoptera: Coccinellidae) when probing the wax filaments produced by its prey (the soft scale *Eupulvinaria hydrangeae* Steinweden (Homoptera: Coccidae), and of the mealybug *Planococcus citri* Risso (Homoptera: Pseudococcidae), are the signals inducing the search for oviposition sites [16]. Oviposition-deterring pheromone is associated with the abundant wax filaments produced by the conspecific larvae of *C. montrouzieri* [17].

*Scymnus nubilus* Mulsant (Coleoptera: Coccinellidae) is a minute ladybird and is very common and dominant aphidophagous predator in the Azores archipelago (Portugal) [8,18], which is located in the North Atlantic, roughly between the coordinates 37° to 40° N latitude and 25° to 31° longitude. The occurrence of this predator is usually associated with aphids in natural habitats and agroecosystems, including orchards and corn fields [19]. Under an optimal temperature (25 °C) and fed an essential prey, *Rhopalosiphum padi* (L.) (Hemiptera: Aphididae), pre-imaginal development takes approximately 18 days and larvae undergo four larval instars before pupating and originating adult females heavier than males (1.37 ± 0.03 mg vs. 1.05 ± 0.02 mg) [20]. Adult females mature 4.8 ± 0.2 days after emergence and lay around 1000 eggs throughout their lifetime [21]. Under laboratorial conditions, longevity was found to be quite large, 150.5 ± 26.6 days for females and more than 6 months for males [20]. Later, it was determined the generation time was 43.6 ± 0.48 days [21]. The only other study on *S. nubilus* found in the bibliography, indicates that fed *Aphis gossypii* Glover (Homoptera: Aphididae) and at 20 °C, the predator pre-imaginal development was longer, 27.3 ± 1.39 days, and females laid 428.2 ± 92.33 eggs during its lifetime (133.2 ± 19.46 days) with a generation time of 51.74 days being determined [22].

Larvae *Scymnus nubilus* are covered by a wax layer produced by dorsal epidermal cells [5]. The wax layer can be easily removed by abrasion and its restoration takes about 24 h and can be restored during any of the developmental larval stages [4]. This morphological defensive mechanism is found in several insect orders (e.g., Homoptera, Coleoptera and Hymenoptera [5]), and has been associated with protection against attacks by predators or parasitoids [15,23,24], balancing their apparent absence of chemical defenses. Indeed, very rarely, older larvae of *S. nubilus* showed reflex bleeding when stressed (personal observations). Other functions, such as protection against UV radiation and reduction of water transpiration, have also been attributed to waxes [4,5]. Their sticky nature not only makes the larvae difficult to bite into but also serves as an entangling agent. Several *Scymnus* species have been observed physically clogging the mouthparts of aggressive ants [4,5,15,25]. Furthermore, there is evidence that larval wax also acts as a mimetic mechanism, allowing those specimens to not be recognized as a threat by aphids [26]. This has been found in some species feeding on mealy aphids attended by ants. Schwartzberg [25] tested if the wax structures of *Scymnus louisianae* Chapin acted as a defense mechanism against *Lasius neoniger* Emery (Hymenoptera: Formicidae). The results showed that the wax attenuated ant aggression towards the ladybird beetle (compared to denuded larvae). From an evolutionary point of view, the success of predatory Coccinellidae is linked to the successful exploitation of ant-attended insects as a food source and then to the development of efficient defensive mechanisms such as larval waxes [27].

As a member of the aphidophagous guilds, *Scymnus nubilus* is strongly subject to an array of biotic interactions. For example, intraguild predation (IGP) is a type of interaction that occurs between species that share a common prey resource. IGP is a form of predation and also an extreme form of competition that can have various ecological effects, such as the alteration of the distribution, abundance, and evolution of the species involved [13,28,29]. For example, it has been shown that *Scymnus posticalis* Sicard has a negative impact on emerging parasitoid numbers from aphid colonies attended by ants [30]. Besides the protection given by the waxes against ant attacks, any additional protection against intraguild predators (IG predators) would be beneficial to the ladybird beetle. Few studies have focused on the defensive potential of the *Scymnus* waxes against IG predators. Völk and Vohland [15] studied this subject regarding the carabid beetle *Platynus dorsalis* Pontoppidan (Coleoptera: Carabidae), and Agarwala and Yasuda [5] with the syrphid larvae *Eupeodes freguens* Matsumura (Diptera: Syrphidae). These studies have shown that waxes effectively conferred protection against IGP.

The objective of this study was thus to evaluate the costs and benefits of wax production in *S. nubilus*. According to life history evolution theory, increased performance in one trait entail a decrease in another trait, which is emphasized when living organisms face stressful conditions [31,32,33]. We observed, under laboratory conditions, a small number of *S. nubilus* waxless larvae, a very rare phenotype/genotype. Thus, the wax production seems to be a heritable trait. Accordingly, we predict that wax production by larvae is a trait selected by adaptive evolution where some benefits (eventually protection against intraguild predation) were traded with some costs. The constant removal of the larval waxes will increase the rate of physiological responses of larvae (e.g., changing the growth rate and/or development time) and thus emphasizing the potential trade-offs. We formulated the following hypothesis: there is a trade-off in wax producing ladybeetles between the protection conferred by wax cover and the physiological or behavioral costs associated with its production. We predict that (1) wax production is an efficient defensive mechanism (against intraguild predation), (2) wax production is associated with detrimental physiological (growth, reproduction) or behavioral effects (behavioral compensation: increased biomass consumption).

## 2. Material and Methods

### 2.1. Insect Rearing

Larvae and adults of *S. nubilus* collected during summer season from corn fields of the Azores, were kept and reared in 2 L plastic cages at 20 ± 1 °C, 75 ± 5% RH and under a light regime of 16L:8D. A mixed diet of *Myzus persicae* Sulzer and *Aphis fabae* Scopoli (Hemiptera: Aphididae) reared on broad bean plants (*Vicia faba* L. Var. Major; Fabaceae) was provided *ad libitum*. Old plants were replaced by fresh ones infested with aphids, every two days. Once a week, larvae and adults were transferred into new rearing cages and the eggs were separated into another container until larval eclosion [34].

*Chrysoperla agilis* Henry, Brooks, Duelli and Johnson (Neuroptera: Chrysopidae) adults were collected during summer season, from corn fields of the Azores. This sprecies is the most important co-occurring predator along with *S. nubilus* in corn fields and apple orchards in the Azores [19,21], and therefore a potential intraguild prey and/or IG predator for *S. nubilus*. They were reared in net cages at 20 ± 1 °C, 75 ± 5% RH and under a light regime of 16L:8D. Adults were fed with a mixture of honey, yeast, and pollen grains dissolved in water. After the emergence of larvae, the same prey species used for *S. nubilus* were provided in broad bean plants until adult emergence. Once a week, old and new adults were transferred into a new cage.

### 2.2. Development Time, Adult Body Weight and Ovarioles Number

To determine the existence of potential trade-offs among life history traits of *S. nubilus* females, development time, adult body weight, and ovarioles number were examined.

Twenty-five couples of *S. nubilus* were separated into 5 cm diameter × 2 cm height rearing plastic boxes (5 couples per box). Adults were allowed to reproduce for 24 h in order to obtain fresh eggs. The adults were then removed, and the eggs were observed twice a day until eclosion of larvae. The newly hatched larvae (n = 48; 9 or 10 from each of the rearing plastic boxes) were individually kept in 3 cm diameter × 1 cm height plastic boxes. This methodological procedure allowed to increase genetic variability of larvae.

This experiment had three treatments, with 16 replicates each (n = 48): (i) wax, (ii) waxless and (iii) stressed individuals with wax. In the former, larval wax was never removed whereas on the waxless treatment, the waxes were removed artificially once a day from hatching until pupation with a fine hair paintbrush Pelikan (#1). Waxes have a secretory nature, produced by dorsal epidermal cells [5], restored within 24 h, and are very smooth, its full removal is a very simple and rapid operation, taking roughly one minute. In the latter treatment, stress was induced on larvae for roughly one minute (which corresponds to the time required for the removal of waxes from one larva), pushing the larva in several directions, including against the walls of the plastic box, using the same fine hair paintbrush Pelikan (#1) but without removing the waxes. Pilot observations did not reveal any kind of skin injuries and negative impact on general activities (e.g., walking and feeding, data not presented). All the larvae from these experiments were checked twice a day at 9 a.m. and 4 p.m. for the presence of exuviae. The development time from egg to adult was recorded.

The adults obtained were weighed 12 h after emergence and sex was determined according to the coloration of the head: yellowish for males and predominantly dark brown for females [35]. After 5 days, *S. nubilus* females (n = 16) were dissected in order to determine the number of ovarioles. All weighings were done using a 10^−7^ mg Sartorius SE 2 ultramicrobalance, Goettingen, Germany.

### 2.3. Behavioral Compensation: Feeding Parameters

To quantify the impact of wax production in biomass consumption of larvae and allocation to growth, four feeding experiments were performed using 12-h old 4th instar larvae, obtained as previously described. Four treatments were considered, with 16 replicates each:

WAX: larvae whose waxes were not removed (not stressed),

WAXLESS: larvae highly stressed (daily removal of waxes),

WAXLESS ONCE: larvae intermediately stressed (removal of waxes only once before transferring the larvae to the arenas with aphids),

STRESSED: larvae daily stressed without removing waxes.

Larvae were isolated and starved for 12 h before conducting the tests. Larvae were provided with 40 *M. persicae* apterous females for 24 h. Both larvae and aphid were weighed before and after the test. In order to determine the weight loss due to dehydration, 40 *M. persicae* were confined in 5 cm plastic boxes (3 cm diameter × 1 cm height) during 24 h Aphids were weighed before and after the referred period. Dehydration was assessed in order to accurately determine only the amount of biomass ingested by *S. nubilus* discounting aphid evapotranspiration.

Biomass consumption (*BC*), weight gain (*WG*) and conversion efficiency (*CE*(%)) were calculated as Borges [20]:

*BC* = *PW_i_* − *PW_f_* − *PW_d_* (1)

*WG* = *LW_f_* − *LW_i_* (2)

(3) $$CE\left(\%\right)=\frac{\left(LW_{f}-LW_{i}\right)}{BC}\times 100$$

where *PW_i_* and *PW_f_* are the initial and final prey weights, *PW_d_* is aphid weight loss due to dehydration. *LW_i_* and *LW_f_* are the larval initial and final weights respectively.

### 2.4. Intraguild Predation

To test if the presence of waxes of larvae of *S. nubilus* provide an effective defensive morphological structure against IGP exerted by *C. agilis*, one experiment comprising two treatments was made: (i) wax and (ii) waxless larvae. For the first experimental treatment, waxes were not removed from larvae of *S. nubilus* and for the second experimental treatment, waxes were removed but only once before confrontation with *C. agilis*. Behavioral observations of the individuals of both treatments, before the tests, did not show differences among wax and waxless larvae (e.g., walking and feeding capabilities).

The developmental stages of the predators (*S. nubilus* and *C. agilis*) were selected according to their body weight so as not to be a factor conditioning the intraguild predation direction. The selected instar for *S. nubilus* was the fourth (0.91 ± 0.09 mg) and the second for *C. agilis* (0.93 ± 0.07 mg). Moreover, no statistical differences were found in the body weight of the larvae of *S. nubilus* used on the wax (*t*-test: *t* = 0.186, *p* > 0.05) and waxless treatment (*t*-test: *t* = −1.267, *p* > 0.05).

Before predator confrontations, the larval stages were produced as follow: eggs of *S. nubilus* and *C. agilis* were separated daily from rearing plastic boxes (see previous description) and transferred to 5 cm × 1 cm plastic boxes. After molting into the selected instars, larvae were fed for 12 h with apterous aphids followed by a period of 24 h of starvation to empty the alimentary canal. Then, *S. nubilus* and *C. agilis* larvae were weighed and placed together in a 5 cm × 1 cm Petri dish and kept inside of the Petri dish for 24 h. After this period, the predators were examined under a stereoscope to determine if they were alive or not. For each of the two IGP treatments (wax or waxless larvae), 15 replicates were carried out).

For each treatment, a test control to estimate the survival rate was performed. Fifteen larvae of each species were isolated individually in a Petri dish for 24 h.

IGP levels were estimated from the rates of predation for *S. nubilus* ($RP_{sn}$) and *C. agilis* ($RP_{ca}$), which were calculated as follows [29]:

(4) $$RP_{sn}=\frac{\left(P\left(ca,sn\right)\times SR_{ca}\right)}{N}\times 100$$

(5) $$RP_{ca}=\frac{\left(P\left(sn,ca\right)\times SR_{sn}\right)}{N}\times 100$$

where “P(ca,sn)” = number of individuals of *C. agilis* killed, “P(sn,ca)” number of individuals of *S. nubilus* killed, “$SR_{ca}$” survival rate of *C. agilis* in control, “$SR_{sn}$” survival rate of *S. nubilus* in control and “N” number of replicates. Survival rates were calculated from the control tests.

The symmetry index of Lucas et al. [28] was used. This index expresses the number of replicates in which a given predator was eaten over the total number of replicates where IGP occurred.

### 2.5. Statistical Analysis

One-way ANOVA followed by LSD post-hoc comparisons was used to compare adult weight, the number of ovarioles of wax, waxless, and stress individuals, biomass consumption, and weight gain of wax, waxless, waxless once, and stress individuals. Data normality and variance homogeneity were assessed by the Kolmogorov–Smirnov and Levene’s tests, respectively. Due to the non-homogeneity of variances, the time of development and the conversion efficiency was compared using the Kruskal–Wallis test and post-hoc comparisons were performed with the Mann–Whitney with the Bonferroni correction. All data were analyzed with SPSS (2020) statistical package version 27.0.1.0. [36].

For the IGP study, the symmetry indices for each combination were compared with the theoretical index of 50% corresponding to a symmetrical interaction, using a Chi-squared test [13,36]. The symmetry index allows us to establish if the interaction is unbalanced in favor of one of the predators. The strength of the IGP between the two species for a given combination was assessed using the *χ*^2^ and was considered (i) symmetrical, when the *χ*^2^ value was not significant, which indicated that the rate of predation of the two species was similar, (ii) asymmetrical, when the *χ*^2^ value was significant and (iii) not significantly asymmetrical, when the *χ*^2^ value was not significant but the rate of predation of the two species differed.

## 3. Results

### 3.1. Development Time, Adult Body Weight and Ovarioles Number

**Figure 1:**
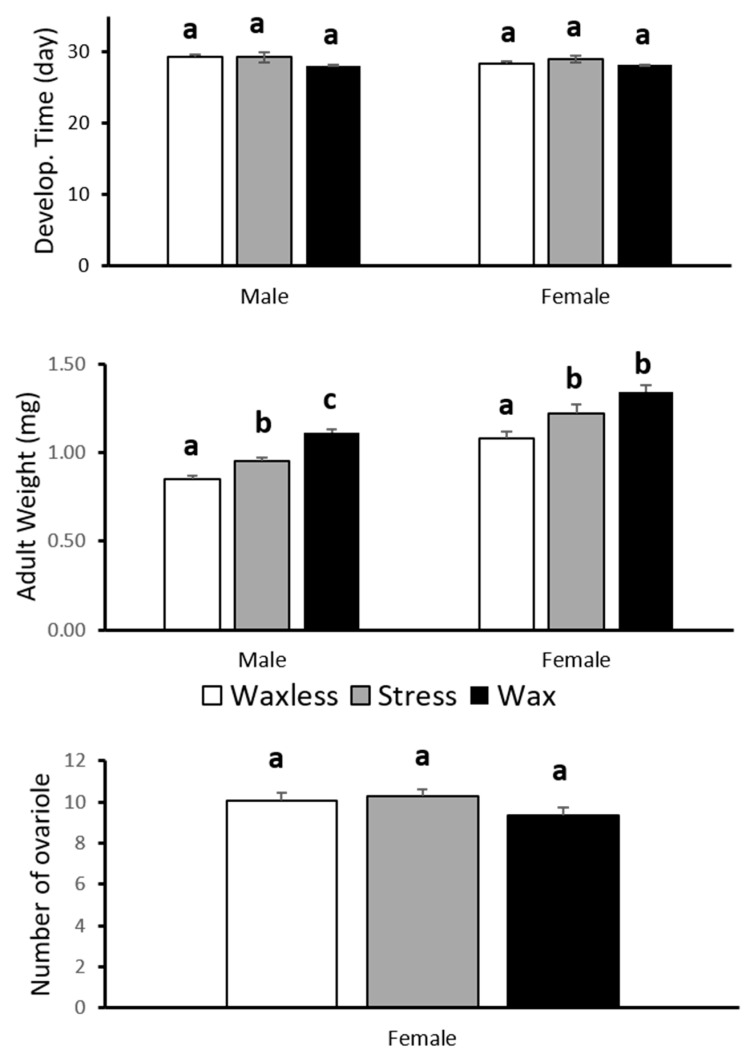
Pre-imaginal developmental time, adult body weight and ovarioles number (mean ± SE) from Wax, Stressed and Waxless *S. nubilus.* Different letters indicate significant differences among treatments (*p* < 0.05).

There was a significant difference for adult weight at emergence between treatments, either for female (F_2,48_ = 9.613, *p* ≤ 0.0001) and male (F_2,48_ = 31.175, *p* ≤ 0.0001) (Figure 1). Adult weights were significantly higher when no waxes were removed during the development of the larvae. No significant differences were found in development time between treatments for females (*χ*^2^ = 0.929, df = 2, *p* = 0.628) and for males (*χ*^2^ = 5.140, df = 2, *p* = 0.077). No significant differences in the ovarioles number were found between treatments (F_2,48_ = 1.745, *p* = 0.187) (Figure 1).

### 3.2. Behavioral Compensation: Feeding Parameters

**Figure 2:**
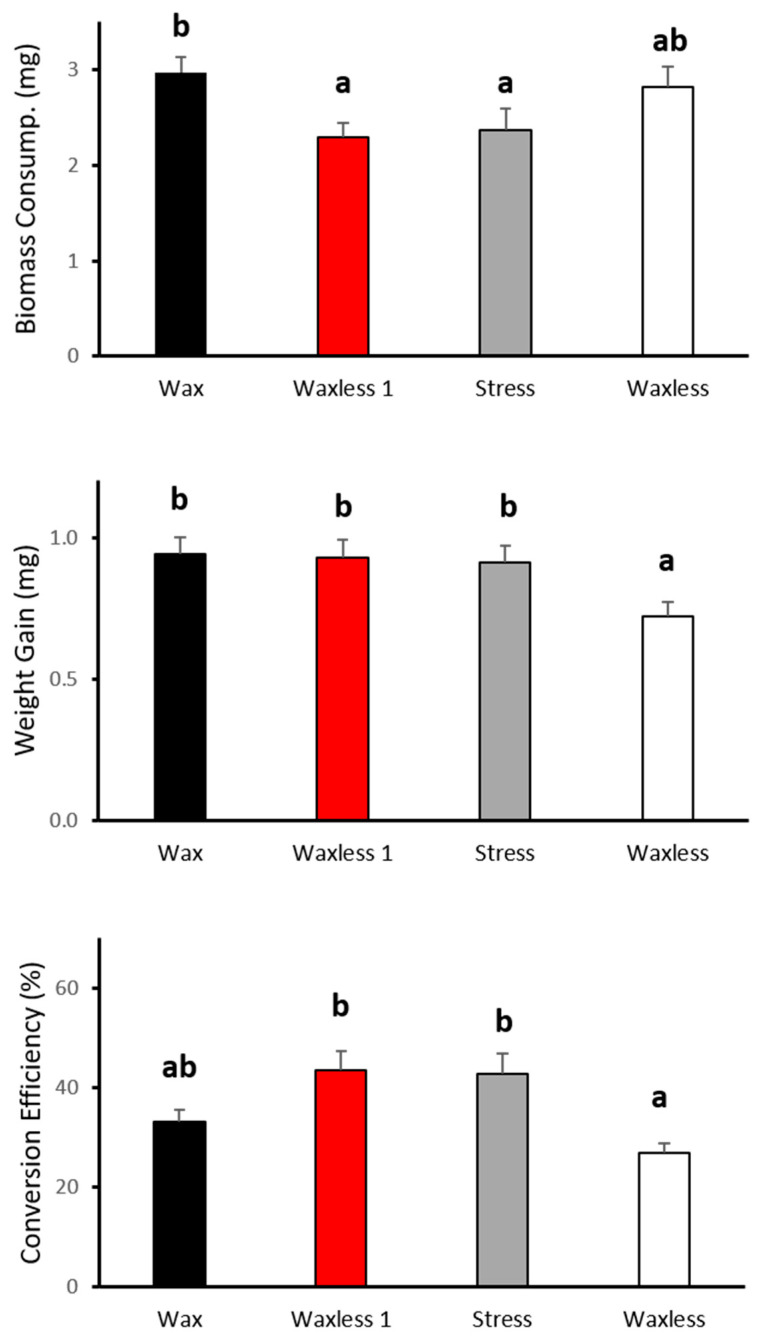
Larval feeding parameters (mean ± SE) from Wax, Waxless once (Waxless 1), Stress and Waxless larvae of *S. nubilus.* Different letters indicate significant differences (*p* < 0.05).

Significant differences were found for biomass consumption (F_3,64_ = 2.905, *p* = 0.042), weight gain (F_3,64_ = 3.403, *p* = 0.023) and conversion efficiency (*χ*^2^ = 18.290, df = 3, *p* ≤ 0.0001) among treatments (Figure 2). Wax larvae ingested more food than stress and waxless-once larvae, but the same amount of food when compared to the waxless larvae. However, waxless larvae gained significantly less body weight than larvae of the other treatments, with the exception of larvae with wax, and, consequently, had lower conversion efficiency (F_3,64_ = 2.905, *p* = 0.042) and similar to larvae with wax (Figure 2).

### 3.3. Defensive Value against Intraguild Predation

**Figure 3:**
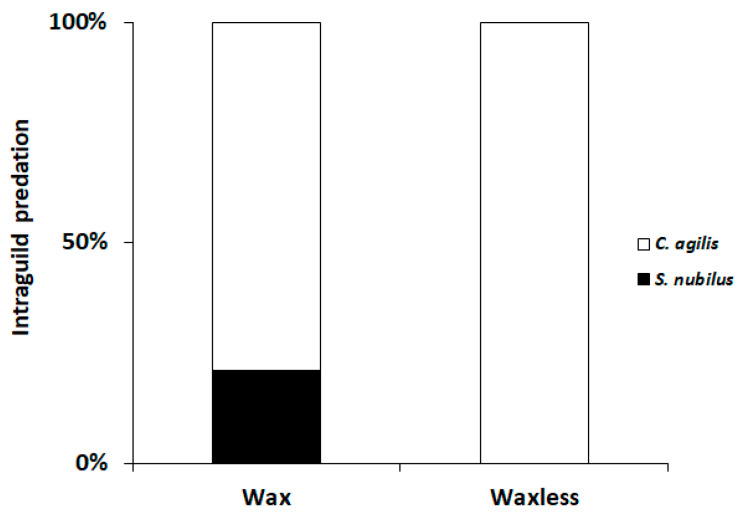
Rate of intraguild predation (RP) by the fourth instar of *S. nubilus* and second instar of *C. agilis.*

During the tests, 100% of the waxless larvae of *S. nubilus* were preyed upon by the IG predator *C. agilis,* whereas the rate of predation decreased to 79% in the test with the wax *S. nubilus* larvae (Figure 3). In a single replicate (out of fifteen) of the wax treatment, both larvae, *C. agilis* and *S. nubilus*, survived. There was a significant asymmetrical IGP in the combination *C. agilis* versus waxless *S. nubilus* (*χ*^2^ = 0.0001, df = 1, *p* = 0.0008). No significant asymmetry was observed for the wax treatment and *C. agilis* (*χ*^2^ = 0.037, df = 1, *p* = 0.15).

## 4. Discussion and Conclusions

The results supported our hypothesis that there is a possible trade-off between costs of wax production and benefits of protection by wax cover against predators. As expected, the larval wax cover decrease the rate of intraguild predation being a defensive mechanism against the lacewing *C. agilis*. Lacewing larvae are recognized as highly aggressive predators commonly involved in intraguild predation [13,28]. However, in the extreme conditions of direct confrontations in Petri dishes, during 24 h, without available preys, wax presence significantly reduced the susceptibility of *S. nubilus* to IGP. More than just a short-term mechanism conferring protection against IGP by lacewings, wax production might be seen as an evolutionary key factor explaining the successful exploitation of aphid colonies by small predators in presence of ants [27], and also of larger competitors (potential intraguild predators). Völkl and Vholand [15] demonstrated that waxes effectively enhanced *Scymnus interruptus* Goeze (Coleoptera: Coccinellidae) survival against the carabid beetle *P. dorsalis*. Agarwala and Yasuda [5] found that *S. posticalis* waxes were also effective against the attack of syrphids.

As predicted, the stress of removing the wax cover in larvae emphasized the potential existence of a trade-off between the investment on wax production and adult biomass. Indeed, some trade-offs are only evident when the organism is under stressful conditions [31,32,33]. The daily artificial removal of wax in the larvae resulted in smaller adults. Nevertheless, females and males of the wax, waxless, and stress treatments showed the same rate of development and reproductive investment in gonads. The number of ovarioles seems to be genetically determined. However, intraspecific variation in ovarioles number can occur and it is positively correlated with body size [37]. Our results showed that despite the costs caused by wax removal on body-weight, the ovarioles number was maintained the same across the treatments. There was, therefore, a reduction of investment in body mass in order to maintain reproductive fitness. Sato et al. [38] also showed that larvae of the ladybird *Harmonia axyridis* Pallas (Coleoptera: Coccinellidae) forced to reflex bleed generated lighter adults compared to the adults developed from larvae that were not stimulated.

Waxless larvae did not increase biomass consumption. Consequently, weight gain seems to be reduced due to the extra cost of producing a wax cover. However, the cost of wax production, translated as biomass, was not evident in a 24-h period (waxless 1 treatment) as well as in the stress treatment. Although the larvae whose waxes were removed only 24 h before conducting the consumption test and the larvae on the stress treatment, consumed less prey biomass, they showed the same increase in weight as the larvae whose waxes were never removed. Conversion efficiency was significantly lower for the waxless treatment, but similar to the individuals conserving the wax. This may indicate that there is a potential shift in the resource allocation caused by wax removal. Our results suggest that there was a resource allocation in wax production overgrowth in the waxless larvae. The organisms must allocate the food resources among metabolism, growth and reproduction [39]. If the energy acquired only covers the metabolic costs then the larvae will disinvest in body growth [40]. Our results suggest that removing the waxes will entail a large resource allocation to regeneration of the cottony cover, leaving less energy available for body growth. Furthermore, fourth instar larvae have greater metabolic costs due to the process of preparation for pupation [40]. Although the larval alimentary physiology was not tested for all instars, fourth instar may be more vulnerable to the stress induced by removing the wax. Indeed, the initial body weight of the fourth instar larva was similar between treatments (F_2,48_ =1.179, *p* = 0.317). This suggests that differences may only arise at the last larval instar.

The small size of *Scymnus* ladybeetles allows the predators to exploit and complete their development on low-density aphid colonies [41,42]. However, small size confers susceptibility to IGP [11,13], on high-density aphid colonies, where larger predators commonly forage. For that reason, smaller predators should rely on specific defensive mechanisms such as wax cover, in order to be able to exploit high-density aphid colonies. Furtive predatory behavior of some small Cecidomyiids and Chamaemyiids also allows them to survive despite the presence of potential IG predators [43,44]. In the case of wax producing *Scymnus* species, wax production should be one of the main factors allowing them to survive in the presence of intraguild predators and ants. Finally, in the case of *S. nubilus,* wax maybe an additional factor explaining why this minute ladybird can thrive in the presence of the aggressive predatory lacewing *C. agilis*.

In conclusion, we found that the absence of the wax cover in larvae of *S. nubilus* turn them more susceptible to intraguild predation by *C. agilis* larvae. Adults originated from waxless larvae were lighter in weight than the ones originating from wax larvae, demonstrating a potential metabolic cost of wax production. Body-weight gain and conversion efficiency were lower in waxless larvae, meanwhile biomass consumption was similar. Results from these two parameters suggested that waxless larvae did not compensate for the physiological cost by eating more aphid biomass. These results indicate the potential existence of a trade-off between growth and protection associated with wax production.
